# Preoperative transarterial embolization of a recurrent orbital solitary fibrous tumor with significant hypervascularity: a case report

**DOI:** 10.1186/s12893-020-01041-x

**Published:** 2021-02-18

**Authors:** Xiawei Wang, Jianqin Shen, Hongguang Cui, Jianwei Pan, Xiaodong Teng, Min Yan, Shi Feng, Wei Ding

**Affiliations:** 1 Department of Ophthalmology, The First Affiliated Hospital, Zhejiang University School of Medicine, Hangzhou, People’s Republic of China; 2 Department of Neurosurgery, The First Affiliated Hospital, Zhejiang University School of Medicine, Hangzhou, People’s Republic of China; 3 Department of Pathology, The First Affiliated Hospital, Zhejiang University School of Medicine, Hangzhou, People’s Republic of China

**Keywords:** Orbital solitary fibrous tumor, Hypervascularity, Transarterial embolization, Operative approach, Case report

## Abstract

**Background:**

Orbital solitary fibrous tumors (SFTs) are rare neoplasms. Recurrent, hypervascular, malignant variations of orbital SFTs have recently been noted and can present a surgical challenge.

**Case presentation:**

We describe a case of a 53-year-old Chinese woman with a history of a resected orbital SFT. She presented with proptosis, limited eyeball movement, and visual loss in the right eye, suggestive of a recurrent SFT. Ocular examination with multimodal imaging revealed a large, nonpulsatile, noncompressible, hypervascular mass behind the eyeball. The patient underwent preoperative transarterial embolization of the main blood supply to the tumor in order to control intraoperative blood loss, followed by ocular enucleation to optimize exposure and enable complete resection of the tumor. Embolization of the right ophthalmic artery and the distal branch of the right internal maxillary artery caused an immediate, substantial reduction of vascular flow, which allowed us to enucleate the eyeball and resect the tumor with minimal blood loss and no complications.

**Conclusions:**

Our case is so far the first Chinese case of successful preoperative embolization of the main blood supply to a large, recurrent, hypervascular orbital SFT. This case also described a different surgical approach to achieve total removal of an orbital SFT without osteotomy.

## Background

Solitary fibrous tumors (SFTs) are uncommon mesenchymal neoplasms that occur most frequently in the pleura and occasionally in the orbit [1]. SFTs are generally considered benign; however, recurrent and malignant variation have been reported. Several studies have reported the highly vascular features of SFTs, which is especially prevalent in recurrent SFTs. Preoperative imaging, including magnetic resonance imaging (MRI) and computed tomography angiography (CTA), are complex assessment tools that may predict hypervascularity [2, 3]. Traditional resection via orbitotomy or craniectomy would carry a risk of uncontrolled bleeding and residual tumors. Embolization is an alternative approach to minimize intraoperative hemorrhage and improve the gross total removal (GTR) rate. There are few reports of transarterial embolization of orbital SFTs; only 4 have been published in English [1, 4–6]. In this study, we successfully resected a large, recurrent orbital SFT with multiple feeder vessels after transarterial embolization. This is the first Chinese case of a successful embolization of 2 main feeder vessels, which also demonstrated a different operative approach to achieve GTR in an orbital SFTs without performing an osteotomy. 

## Case presentation

A 53-year-old Chinese woman presented to the hospital with an 8-year history of progressive proptosis of the right orbit. It is associated with progressive reduced of vision, visual field deficits and excessive tearing. She denied diplopia, eye pain or headaches. Nine years prior to this presentation, she had undergone surgical resection of a right orbital tumor via anterior orbitotomy, and histopathology confirmed that it was an SFT. Nine months post operative, patient began to develop symptoms that reflected a slow increase of tumor size. Because the tumor was closely adherent to the optic nerve, it was thought that resection might injure the nerve, potentially damaging the patient’s sight. Considering her normal best corrected visual acuity, patient was advised to attend regular follow-ups. However 2 years later the patient was no longer heard from until 2018. 

On examination, there was a large non-pulsetile, non-compressible mass in the right orbit cause the proptosed eye displaced infero-temporal. Exopthamometer measurement showed 23 mm proptosis in the right eye and 15 mm in the left eye. The best corrected visual acuity in the right eye was 2/20 with restricted visual field. The cornea is clear, conjunctiva was injected and extra ocular movement was restricted in elevation and adduction. Fundus examination showed significant optic nerve edema. Examination in the left eye and general physical examination were normal.

**Fig. 1:**
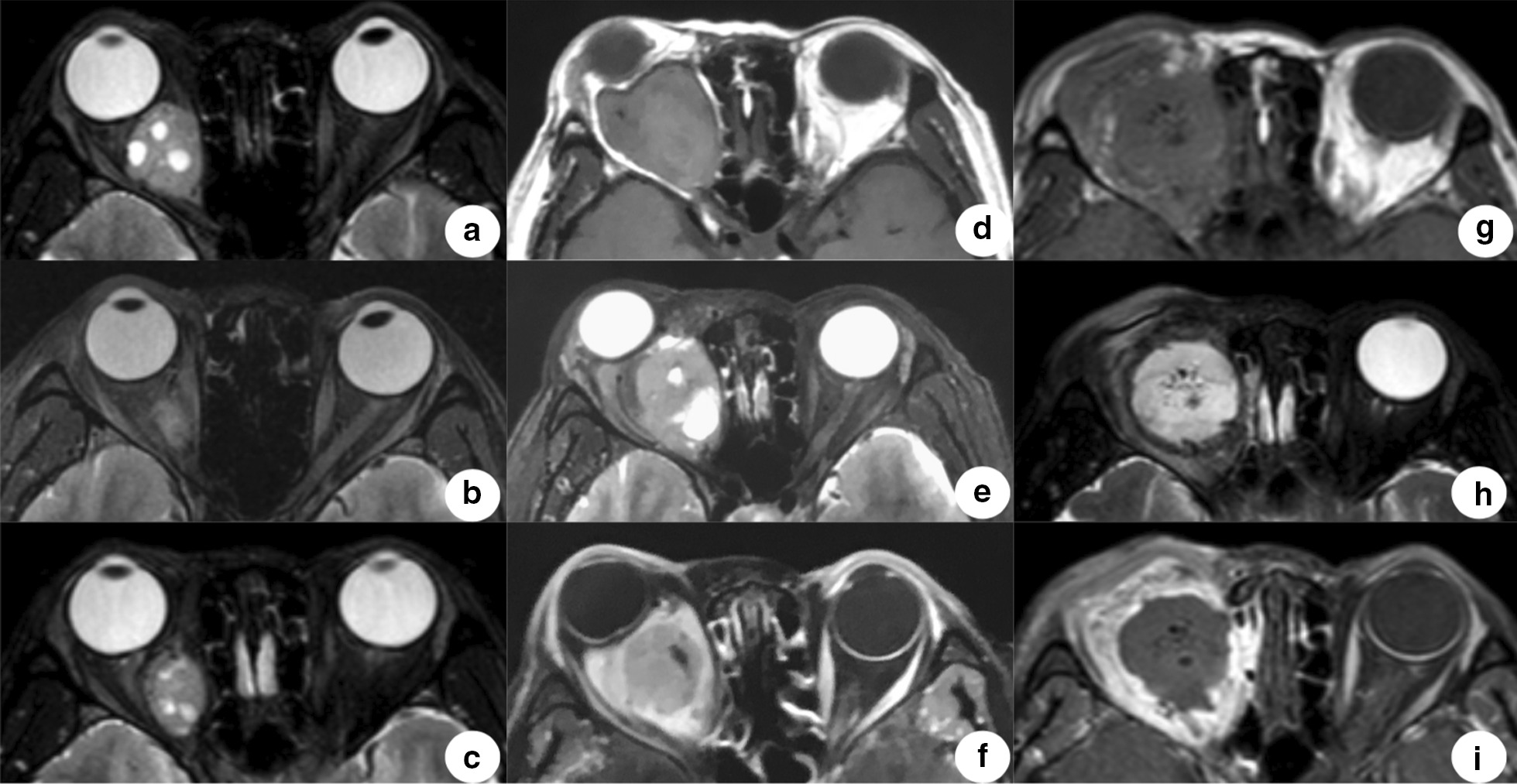
Magnetic resonant imaging. **a** Axial T2 obtained in 2009 shows a well encapsulated and mixed-intensity circular lesion with 3 septate cystic spaces before the first operation. **b** Recurrent lesion 9 months after the initial surgery. **c** Appearance of the lesion in 21 months after the previous surgery. **d**–**f** A large heterogeneous multicystic intraconal mass occupying almost the entire posterior orbit. **g**–**i** The orbit was filled with gelatin sponge, with no visible neoplasm signals 1 week after total resection. **a**, **b**, **c**, **e**, **h** T2 image, **d**, **g** T1 image, and **f**, **i** post-contrast T1 image

Upon reviewing of the MRI in 2009 before the initial resection, an ill-defined, well-encapsulated, mixed-intensity circular lesion measuring 2.4 cm × 2.1 cm with 3 septate cystic spaces was observed posterior to the right orbit between the optic nerve and the medial rectus, iso- or hypo-intense on T1, slightly hyper-intense on T2 (Fig. 1a), and significantly enhanced on contrast image. Unfortunately, 9 months after the initial operation, the lesion proximal to the medial orbital wall reappeared with the same signals (Fig. 1b). Twenty-one months later, the lesion had increased in size to approximately 1.8 cm × 1.5 cm (Fig. 1c). The patient had not followed-up for 7 years since then and came back the clinic in 2018. The MRI was ordered, and the result showed a large, heterogeneous, multi-cystic, 3.4 cm × 3.2 cm intraconal mass occupying almost the entire posterior orbit and causing expansion of the orbital cavity. The signal was mostly iso-intense on T1 images (Fig. 1d), and was slightly hyper- to significantly hyper-intense on T2 images (Fig. 1e). Diffusely heterogeneous enhancement was observed after intravenous gadolinium administration (Fig. 1f), which might represent fast-flow vessels within the tumor. The optic nerve was wrapped and displaced laterally, and the extraocular muscles were not identifiable.

The recurrent SFT was possibly predicted. Color Doppler sonography of the posterior eyeball vessels further indicated an arterial blood flow spectrum with a resistive index of 0.58. CTA further confirmed the marked vascularity and indicated that a high-blood-flow enlarged ophthalmic artery was one of the potential main feeder vessels.

**Fig. 2:**
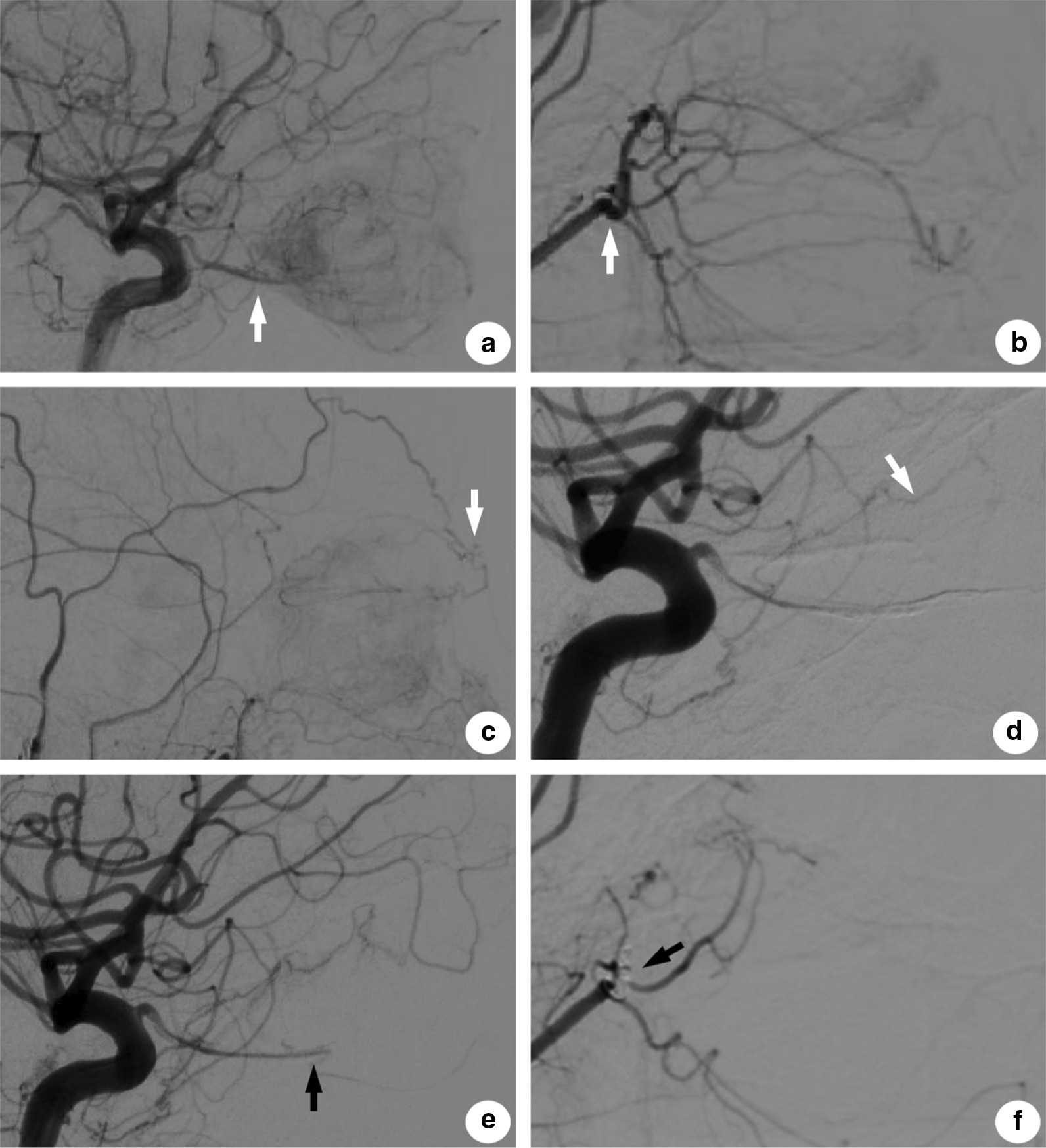
Imaging of the feeder vessels before and after embolization. **a** Multiple feeder vessels arising from the right ophthalmic artery (white arrow). **b** Feeder vessels arising from the distal branch of the right internal maxillary artery (white arrow). **c** Feeder vessels arising from the right frontal branch of the superficial temporal artery (white arrow). **d** Blood supply from the perforation of the right internal carotid artery (white arrow). **e** Injection of Onyx liquid via the ophthalmic artery (black arrow). **f** Postembolization of the feeder vessels arising from the right internal maxillary artery using 2 coils (black arrow)

En bloc tumor debulking utilizing existing techniques presented significant difficulties. The tumor was large, occupying vast majority of the retrobulbar orbital cavity, and was firmly adherent to the right optic nerve with marked vascularity. It was determined that it would be difficult to completely resect the tumor using conventional orbitotomy or craniectomy and preserve the patient’s existing vision at the same time. There was also concern that traditional orbitotomy surgery would cause massive blood loss, which might result in incomplete resection of the tumor, with the possibility of future recurrence or even malignant metastasis [4, 7]. In addition, we were faced with the dilemma of whether to enucleate the eyeball. Considering the patient’s poor vision in that eye and her intention not to undergo an osteotomy, we obtained the patient’s written consent to perform an ophthalmectomy to optimize exposure of the lesion in order to resect it as completely as possible, after first performing preoperative embolization of the blood supply to the tumor to control intraoperative hemorrhage. Angiography demonstrated that the majority of the vessels feeding the tumor arose from the right ophthalmic artery (Fig. 2a) and the distal branch of the right internal maxillary artery (IMA) (Fig. 2b). The right frontal branch of superficial temporal artery (Fig. 2c) and the perforation of the right internal carotid artery (ICA) (Fig. 2d) also contributed to the tumor’s blood supply. Because the entry points of the feeder vessels were too thin for the microcatheter (ASAHI Inc., Aichi-ken, Japan) to pass through, the only alternative path was via the right ophthalmic artery. To devascularize the vessels originating from the right ophthalmic artery, ethylene vinyl alcohol copolymer (Onyx) liquid embolic agent was injected into the feeder branches and the central retinal artery (Fig. 2e). Other main feeder vessels arising from the right internal maxillary artery were also embolized using 2 coils (Fig. 2f). Vascular flow was markedly reduced immediately following the embolization procedure.

Approximately 1 h after embolization, the patient experienced 4 significant phenomena: (1) extreme pain in the right eye; (2) immediate loss of light perception; (3) significant swelling of conjunctiva; and (4) a decrease in heart rate of 45–50 bpm compared with the previous heart rate of 65–80 bpm. Of the 4 phenomena observed after embolization, the drastic drop in heart rate was especially alarming. ‘Oculo-cardiac reflex’ was diagnosed, so 1 mg intravenous atropine was administered preoperatively, which stabilized the patient’s heart rate at 60–92 bpm, and surgical tumor resection was completed as scheduled.

The eyeball was extracted firstly. The giant mass appeared slightly reddish internally, with a wide engorged vein on the central surface. It filled the orbit and displaced the retro-bulbar segment of the right optic nerve, shifting it infero-temporally. Atrophy of the orbit tissues was also noticed. The tumor capsule was found to adhere to the periosteum of the medial orbital wall. As a result, debulking was performed to isolate the neoplasm, followed by en bloc tumor resection. The total volume of blood loss was less than 50 mL, and there was no abnormal bleeding. After the tumor was resected, the patient’s heart rate stabilized at 60–80 bpm. An ocular prosthesis was made for aesthetic purposes. One week postoperatively, an MRI demonstrated that the orbit was filled with gelatin sponge that was slightly hyper-intense on T1 (Fig. 1g) and T2 (Fig. 1h) without enhancement in the post-contrast image (Fig. 1i). Further radiotherapy and formal orbital prostheticrehabilitation for the patient was planned.

**Fig. 3:**
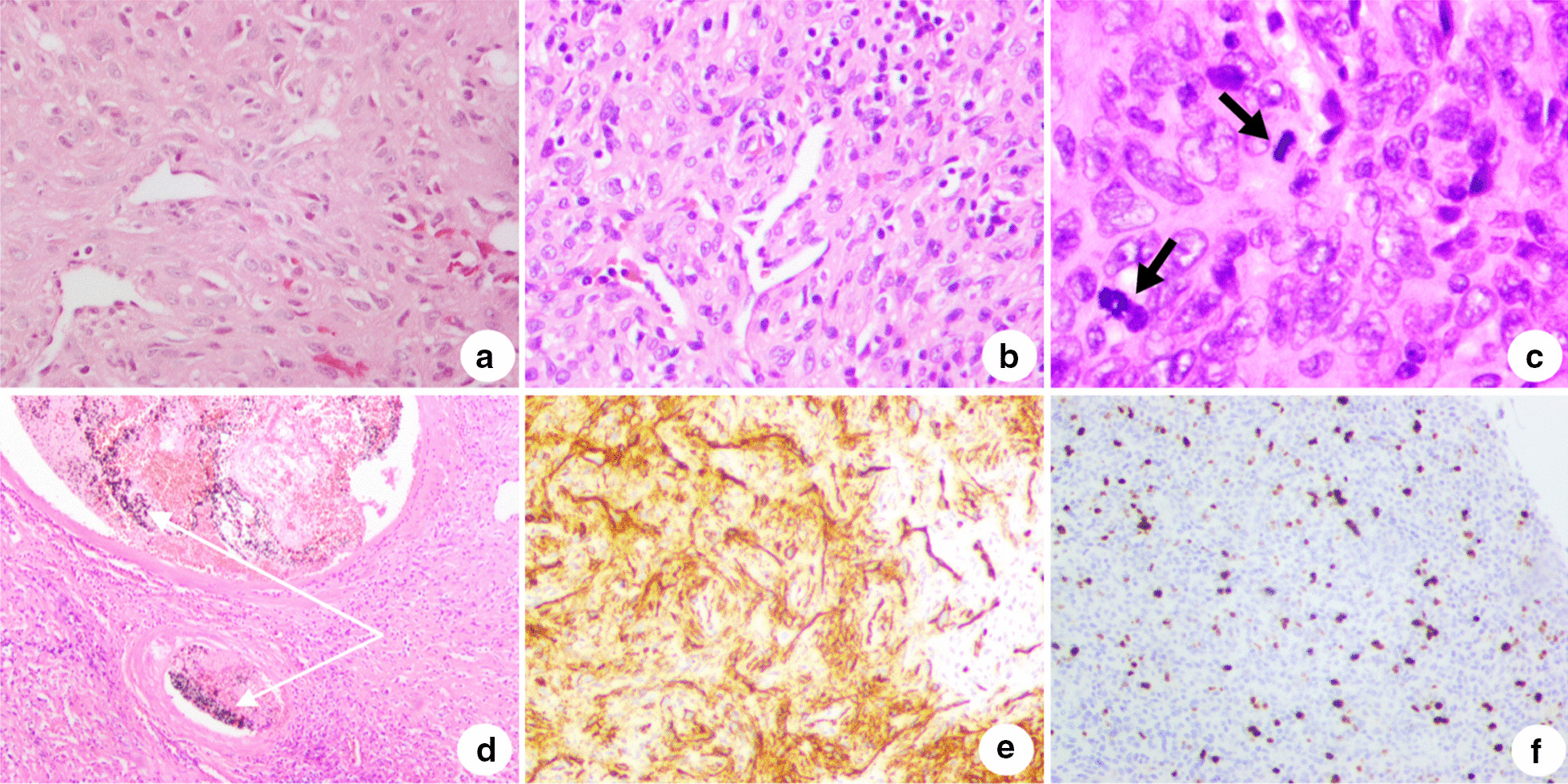
Histopathological and immunohistochemical staining of the tumor. **a** Diffuse spindle cells with ‘anter’-like vessels on background (HE staining; original magnification: × 200). **b** Haphazardly arranged heterotypical spindle cells with variable collagenous and ‘staghorn’ vessels in the background (HE staining; original magnification: × 200). **c** Frequent mitoses (HE staining; original magnification: × 400). **d** Onyx embolization material with the appearance of granular black pigment (tantalum powder) (white arrows) in the center of the vessels (HE staining; original magnification: × 50). **e** Tumor cells positive for CD34 (original magnification: × 200). **f** Tumor cells positive for Ki-67 (approximately 15%) (original magnification: × 200). *HE* hematoxylin and eosin

Pathological review of the tumor following the initial surgery in 2009 showed that the tumor was composed of diffused spindle cells, with oval to fusiform nuclei and a pale eosinophilic cytoplasm. ‘Antler’-like vessels were noted in the background (Fig. 3a). The immunohistological study showed that it was positive for cell differentiation 34 (CD34) and negative for cytokeratin (CK), smooth muscle actin (SMA), epithelial membrane antigen (EMA), and S-100 protein. These findings are consistent with the diagnosis of SFT. Histopathological examination of the tumor following the second operation revealed haphazardly arranged spindle cells with more heterotypical changes than were found in the previous sample. The background stroma was variably collagenous and rich, with the presence of staghorn-shaped vessels (Fig. 3b). Frequent mitoses were found > 4/10 high-power fields (HPF) (Fig. 3c). In addition, a low-power view of the tumor showed the Onyx embolization material appearing as small, granular, black pigment (tantalum powder) in the feeder vessels (Fig. 3d). Immunohistologically, the tumor cells exhibited diffuse immunoreactivity for CD34 (Fig. 3e), and CD99 and was approximately 15% positive for Ki-67 (Fig. 3f).

## Discussion

SFTs are rare spindle cell tumors first described in 1931 in pleural tissue [8]. Since then, researchers have found these tumors in extrapleural locations such as the lungs, liver, kidneys, thyroid gland, parotid gland, pericardium, peritoneum, mediastinum, nasal cavities, and orbit [9]. Clinically confirmed orbital SFTs are very rare, with approximately only 90 cases reported up to date [4]. These tumors are generally considered benign but recurrent and malignant variations have recently been noted. 

Since recurrent or malignant SFTs can be highly vascular with multiple large feeder vessels, the attempts to biopsy and resection them might fail because of massive hemorrhage without taking preoperative measures [4, 7]. Preoperative embolization is recommended to reduce the risk of hemorrhage. Matthew et al. first performed percutaneous embolization under fluoroscopic radiographic guidance in 2012 [10]. However, this technique can only be achieved when the lesions are visible. The endovascular approach is more effective for tumors in the orbit. Compared with previous reposts [1, 4–6], we demonstrated a large intraconal mass with multiple feeder vessels arising from 4 points of origin (Table 1), including main feeder vessels with numerous tangled branches arising from the ophthalmic artery and the IMA. Because the points of entry of the vessels from ophthalmic artery were too thin for the microcatheter to pass through, liquid Onyx was an effective means of embolizing these tangled feeder vessels due to its slow precipitation properties [1]. In particular, 2 embolic coils were sufficient to block off the 2 main feeder trunks arising from IMA. The combined use of the Onyx liquid and the embolic coils significantly devascularized the tumor’s blood supply, resulting in minimal total blood loss during the subsequent surgery. Other highly vascular orbital tumors can be treated in the same manner, no matter where the tumor is located within the orbit. It has proved to be an effective method of preoperatively devascularizing and embolizing feeder vessels.

**Table 1:** Summary of reported intraorbital SFTs with preoperative embolization [1, 4–6]

Source	I Jeeva et al	Kaitlyn M et al	Nafiseh H et al	Munehiro D et al	Present case
Year	2013	2014	2015	2017	2019
Country	UK	USA	USA	Japan	China
Age/sex	22/M	58/F	55/F	42/M	53/F
Position	Extraconal superior wall of orbit	Extraconal medial wall of orbit	Extraconal medial wall of orbit	Extraconal medial wall of orbit	Intraconal posterior orbit
Size (mm)	Nm	48 (Diameter)	27 × 20 × 15	30 × 19 × 33	35 × 30 × 22
Shape	Long oval	Long oval	Oval	Long oval	Oval
Recurrence	N	N	N	N	Y
Feeding A	Ophthalmic A	Ophthalmic A; IMA nasal–frontal Br	Ciliary A	Supraorbital A; infraorbital A muscular Br	Ophthalmic A; IMA; STA frontal Br; ICA perforation
Embolic A	Distal ophthalmic A	Distal ophthalmic A	Tumor vessels	Supraorbital A; infraorbital A muscular Br	Distal ophthalmic A; distal IMA
Embolic material	Onyx	NBCA	Onyx	Coil, NBCA	Coil, Onyx
Surgical approach	Lid crease approach	Anterior orbitotomy	Orbitotomy	Craniotomy Endoscopic transnasal	through ophthalmectomy
Blood loss	Nm	20 ml	Nm	180 ml	50 mL
Removal rate	Total	Total	Total	Total	Total
Complications	N	N	N	N	N

*F* female, *M* male, *Nm* not mentioned, *N* no, *Y* yes, *A* artery, *Br* branch, *IMA* internal maxillary artery, *ICA* internal carotid artery, *STA* superficial temporal artery, *NBCA* n-butyl-2-cyanoacryla

Previous reports have used orbitotomy or craniectomy to expose the mass [1, 5, 6]. In these cases, the tumors were mainly located extraconally and on the superior or medial wall of the orbit. As a result, it was possible to preserve the patients’ visual acuity because the optic nerve was seldom involved. Orbitotomy or craniectomy performed by experienced surgeons would not injure the optic nerve and might be suitable for removing medium-sized tumors in the previously mentioned locations. In the current case, the neoplasm was large, retrobulbar, and intraconal. The patient’s previous history and clinical clues enabled us to preoperatively predict that this SFT had the possibility of malignant transformation. Admitting that preoperative embolization successfully minimized the blood loss, surgical exposure using orbitotomy or craniectomy combined with osteotomy to receive GTR would have the potential to cause more complications and aesthetic deficits [11]. Moreover, recent reports indicate that endoscopic endonasal intraorbital surgery (EEIS) can be an effective surgical approach for excising orbital tumors [4, 12]. The EEIS technique was applicable in this case because the mass was localized adjacent to the medial wall. However, recurrent SFTs might be malignant and invasive, and resecting them under limited visualization and in a piecemeal manner may cause uncontrolled bleeding and leave residual tumor tissue. Considering the patient’s poor vision and her willingness, we obtained the patient’s written consent to perform an ophthalmectomy to optimize exposure of the lesion with few complications. 

In this case, embolizing the tumor’s main blood supply caused an immediate cascade of reactions, including intense eye pain, loss of vision, conjunctival edema, and a decrease in heart rate. The eye pain may have been the result of acute ischemia or vasospasm of the tumor, or the cytotoxicity of dimethyl sulfoxide adjuvant used in Onyx. Conjunctival edema secondary to circulatory disturbance and inflammatory reaction likely raised intraorbital pressure in the eyeball, which further aggravated the pain [13]. However, the associations between these phenomena remain to be investigated. Loss of light perception could possibly have been the result of embolization of the neoplasm’s main blood supply and arteria ophthalmica. The decrease in the patient’s heart rate and its immediate return to normal levels after resection may be an example of the ‘oculo-cardiac reflex’. Heart rate variability before, during, and after the resection requires further investigation. 

In conclusion, orbital SFTs can be hypervascular, especially in recurrent and invasive cases. Preoperative CTA can be performed to assess the blood supply to the lesion. Embolism can be an effective technique to minimize intraoperative hemorrhage. The size and location of the tumor, patient visual function, as well as patients’ intention should be considered when deciding the surgical approach. The surgical techniques described in this study could be a feasible approach for complete resection of orbital SFTs.

## Data Availability

All data generated or analysed during this study are included in this published article.

## Abbreviations

SFTs: Solitary fibrous tumors
MRI: Magnetic resonance imaging
CTA: Computed tomography angiography
GTR: Gross total removal
IMA: Internal maxillary artery
ICA: Internal carotid artery
CD: Cell differentiation
CK: Cytokeratin
SMA: Smooth muscle actin
EMA: Epithelial membrane antigen
HPF: High-power fields
EEIS: Endoscopic endonasal intraorbital surgery
